# XSI—a genotype compression tool for compressive genomics in large biobanks

**DOI:** 10.1093/bioinformatics/btac413

**Published:** 2022-06-24

**Authors:** Rick Wertenbroek, Simone Rubinacci, Ioannis Xenarios, Yann Thoma, Olivier Delaneau

**Affiliations:** School of Management and Engineering Vaud (HEIG-VD), HES-SO University of Applied Sciences and Arts Western Switzerland, Yverdon-les-Bains 1401, Switzerland; Department of Computational Biology, University of Lausanne, Lausanne 1015, Switzerland; Department of Computational Biology, University of Lausanne, Lausanne 1015, Switzerland; Department of Computational Biology, University of Lausanne, Lausanne 1015, Switzerland; School of Management and Engineering Vaud (HEIG-VD), HES-SO University of Applied Sciences and Arts Western Switzerland, Yverdon-les-Bains 1401, Switzerland; Department of Computational Biology, University of Lausanne, Lausanne 1015, Switzerland

## Abstract

**Motivation:**

Generation of genotype data has been growing exponentially over the last decade. With the large size of recent datasets comes a storage and computational burden with ever increasing costs. To reduce this burden, we propose XSI, a file format with reduced storage footprint that also allows computation on the compressed data and we show how this can improve future analyses.

**Results:**

We show that xSqueezeIt (XSI) allows for a file size reduction of 4-20× compared with compressed BCF and demonstrate its potential for ‘compressive genomics’ on the UK Biobank whole-genome sequencing genotypes with 8× faster loading times, 5× faster run of homozygozity computation, 30× faster dot products computation and 280× faster allele counts.

**Availability and implementation:**

The XSI file format specifications, API and command line tool are released under open-source (MIT) license and are available at https://github.com/rwk-unil/xSqueezeIt

**Supplementary information:**

Supplementary data are available at *Bioinformatics* online.

## 1 Introduction

Generation of genotype data has been exponentially growing over the last decade. Projects including hundreds of thousands of participants with genotype data coming from SNP arrays, whole-genome or exome sequencing (WGS/WES) at hundreds of millions of genome loci are becoming more common. The use of data from large cohorts has become instrumental for disease research (Gudmundsson *et al.*, 2021; Morris and Cardon, 2019; Sudlow *et al.*, 2015; Visscher *et al.*, 2017) and population genetics (Nait Saada *et al.*, 2020). Large sample sizes combined with WGS/WES technology allow the analysis of rare variants to detect small effects with higher statistical power, leading to novel discoveries. Large datasets also allow for better haplotype phasing and genotype imputation of new samples, boosting accuracy of downstream analysis methods (Marchini, 2019).

The growth of genotyped cohorts comes with a larger storage footprint as well as increasing loading times. As an example of state-of-the-art WGS datasets, data from the UK Biobank project providing genotypes from WGS contain ∼150 000 samples typed on ∼600 000 000 variants (Halldorsson *et al.*, 2021), the TOPMed project contains ∼50 000 samples with ∼400 000 000 variants (Taliun *et al.*, 2021) and GnomAD contains ∼76 000 samples with ∼700 000 000 variants (Karczewski *et al.*, 2020). The resulting footprints of these datasets are substantial, ∼700 TB of storage for the UK Biobank WGS GATK genotype calls (Halldorsson *et al.*, 2021). The data generated from such projects are becoming excessively large and hard to handle.

The standardly adopted formats for genotype data are the text-based Variant Call Format (VCF), its binary equivalent BCF (Danecek *et al.*, 2011) and PLINK (Chang *et al.*, 2015). Formats that can all be further compressed with standard compression methods such as gzip. However, these formats are not optimal for population scale WGS genotype data. Several formats have been introduced in recent years to reduce the data size (Danek and Deorowicz, 2018; Deorowicz and Danek, 2019; Deorowicz *et al.*, 2013; Durbin, 2014; Layer *et al.*, 2016; LeFaive *et al.*, 2021; Li, 2016; Tatwawadi *et al.*, 2016). Exploitation of sparse representations, reordering of the data and re-encoding allowed file sizes to be reduced by an order of magnitude compared with standard VCF+gzip. However, one of the major issues of novel formats is that they lack application support, as they only come with a compression and decompression tool that allows conversion from and to a common format (e.g. VCF). This saves space for long-term storage but only adds overhead to analyses.

To tackle this issue, in this article, we present xSqueezeIt (XSI), a file format made to store genotype data in a compact representation that allows for fast computation and analysis. Using data from the one thousand genome project (The 1000 Genomes Project Consortium, 2015), the Haplotype Reference Consortium (McCarthy *et al.*, 2016), the UK Biobank (SNP array and WGS) (Bycroft *et al.*, 2018; Halldorsson *et al.*, 2021) and simulated data, we demonstrate the compression performance of XSI, in both file size and access time compared with existing methods. To show how to integrate XSI in an application, file format support was added to SHAPEIT4 (Delaneau *et al.*, 2019). Finally, benchmarks of computation performed directly on the encoded data structures are given to show the benefits of the format in terms of performance for future analyses.

## 2 Materials and methods

XSI relies on a hierarchical block-based compression strategy. The compression hierarchy has two levels. First a (genetic) data specific algorithm is used and second the resulting data are handled as a general purpose file compression problem. In order to achieve fast random access to any variant loci, a block-based approach combined with indexing is used.

**Fig. 1.**
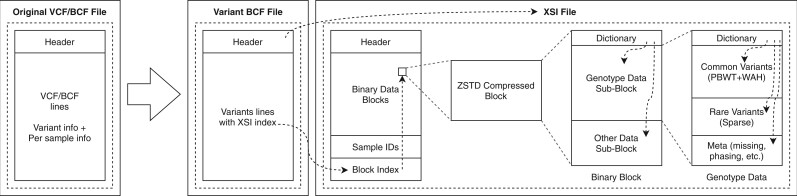
The original VCF/BCF file is split in two files: a BCF file with variant information and a XSI file with per sample information (e.g. genotype data). The BCF can be queried to retrieve the per-sample information with an index referencing the XSI file. The XSI file itself is composed of binary data blocks which hold the sample data for a number of BCF lines, each block being compressed by zstd. The blocks themselves hold a small dictionary referencing their content, for example genotype data. The sub-blocks are independent and compressed with a method specific to the data type. Dashed arrows represent references

Figure 1 shows an overview of a VCF/BCF file and its equivalent XSI representation with internal structure. Variant information is kept in BCF format and holds a reference to the associated XSI file instead of the original genotype (and other) data. This reference is used to query the XSI file and extract the data. The XSI file itself is a collection of binary blocks, which contain a given number (8192 by default) of BCF lines (variant loci) encoded in a way specific to the data type. Blocks hold a dictionary that references its contents and are compressed with Zstandard (zstd) (Collet and Kucherawy, 2018). The file format specifications, internal binary structures and algorithms are available at https://github.com/rwk-unil/xSqueezeIt.

### 2.1 Compression of genotype data

**Fig. 2.**
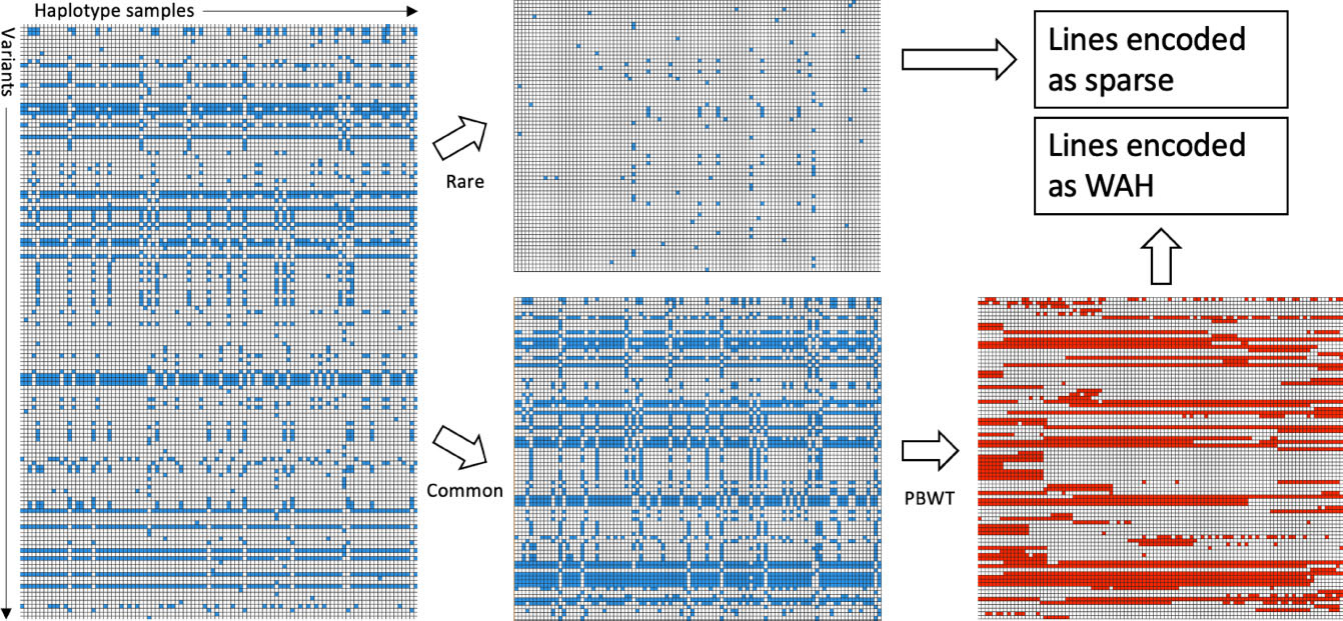
Sketch of data reordering and re-encoding scheme. Input genotype data shown on the left: each column is a sample, each line a variant loci, an empty square is the reference allele, a colored square the alternate allele. The data are split into rare and common variants based on a MAF threshold. Rare variants are encoded as sparse lines. For common variants, each line is reordered given the previous lines, thanks to the PBWT. Reordered lines are WAH encoded

We use two different strategies to compress genotype data, depending on allele frequency. First, for common variants [minor allele frequency (MAF) >0.1%, by default], we exploit linkage disequilibrium (LD) in the population to reorder the data and allow for better compression. This is done by leveraging the positional Burrows–Wheeler transform (PBWT) to reorder the data before re-encoding. This approach has been shown to be effective (Durbin, 2014) and is the main component of many genotype compression algorithms (Deorowicz and Danek, 2019; LeFaive *et al.*, 2021; Li, 2016). After transforming the data through the PBWT, genotype data are encoded using a word-aligned hybrid (WAH) approach (Wu *et al.*, 2001). This is a variation of run length encoding (RLE) that is more robust to patterns of alternating symbols. WAH also allows efficient operations on the encoded data (Wu, 2001). Second, for the rare variants (MAF ≤0.1%), we employ a sparse representation, directly exploiting the fact that the number of samples carrying the variant is small. These two methods are the main contributors to the file size reduction. Figure 2 illustrates the procedure on a few samples from the one thousand genome project (The 1000 Genomes Project Consortium, 2015).

The PBWT is applied on the common variants where it improves compression the most, as applying the transformation to all variant loci would be expensive, especially with large numbers of samples because each locus would require rearrangement of all samples. In order to improve per variant random access performance, the PBWT is recomputed from the initial sample ordering for each block, making each block independent. Therefore, access to data in a block does not require decoding any other block. This improves random access performance at the cost of a slightly bigger file.

The chosen algorithms and encoded representations required to be computationally inexpensive in order to scale with large datasets. Ideally, the compression, access and decompression algorithms should not exhibit worse than linear complexity with relation to the number of genotypes. The sparse representation of XSI allows for computations through numerous existing libraries (e.g. BLAS and libboost) and the PBWT WAH-encoded representation also allows for interesting direct computations, for example, haplotype search and matching (Durbin, 2014; Wu, 2001).

### 2.2 Storage and compression of other fields

The hierarchical format shown in Figure 1 allows the addition of other sub-blocks for storage of other data found in VCF files, such as genotype likelihoods, read count and imputed dosages. Each sub-block can be specifically encoded and can employ different compression strategies. This also makes the format extensible without breaking compatibility. Older versions of the software simply ignore unknown sub-blocks. This also allows for development of sub-block formats by other developers. Sub-blocks are identified by their dictionary ID and developers can ask for a unique non-overlapping ID range through a github issue. This would guarantee that different implementations do not overlap even if their specifications are kept as closed source. This also makes it possible to add encrypted sub-blocks, providing privacy where needed without necessarily encrypting all the data. Finally, this also allows for alternative compression methods on already supported data types.

### 2.3 Compression of encoded blocks

Once the sub-blocks have been re-encoded with their specific compression scheme, further reduction can still be achieved. At this stage, a general purpose compression algorithm is applied to each block. Where VCF/BCF employs bgzip, we apply zstd (Collet and Kucherawy, 2018), a state-of-the-art LZ (Ziv and Lempel, 1977) compressor, because of its compression and decompression speed (lzbench an in-memory benchmark of open-source LZ77/LZSS/LZMA compressors. https://github.com/inikep/lzbench).

## 3 Results

We compared XSI with BCF (Danecek *et al.*, 2011), BGT (Li, 2016), GTC (Danek and Deorowicz, 2018), GTShark (Deorowicz and Danek, 2019), PBWT (Durbin, 2014), SAVVY (LeFaive *et al.*, 2021) and PLINK2 (Chang *et al.*, 2015) in terms of features, file size and conversion times across four gold standard datasets and a simulated region: The 1000 genome project phase 3 (The 1000 Genomes Project Consortium, 2015), the Haplotype Reference Consortium (McCarthy *et al.*, 2016), SNP array genotypes from the UK biobank (Bycroft *et al.*, 2018), WGS genotypes from the UK biobank (Halldorsson *et al.*, 2021) and a 10-Mb region with 1 million samples and over 2 million variants simulated with msprime (Kelleher and Lohse, 2020) (extended information in Supplementary Section S1).

The benchmarks for UK Biobank WGS data have been run on the DNANexus research access platform on Xeon Platinum 8000 series with 64 GB of RAM. All other benchmarks have been run on an Intel Xeon E5-2680v3 CPU with 64 GB of RAM.

### 3.1 Feature support

Alternative file formats usually only implement a subset of all VCF/BCF features. Table 1 summarizes features of XSI in comparison to existing formats.

**Table 1.** Features per file format (details in Supplementary Section S4)

	xsi	bgt	gtc	gtshark	pbwt	savvy	plink2	bcf
Keeps variant info	✓	✗	✗	✓	✗	✓	✓	✓
Mixed ploidy support	1–2	✗	✗	✗	✗	✓	✓	✓
Keeps phase information	✓	✗	✗	✗	✗	✓	✗	✓
Multi-allelic site support	✓	Split	Split	Split	Split	✓	Split	✓
Sample extraction	✓	✓	✓	1	✓	✓	✓	✓
Region extraction	✓	✓	✓	✗	✓	✓	✓	✓
Simultaneous access with VCF files through HTSLIB (sync_reader)	✓	∼	∼	✗	✗	✗	✗	✓

Several file formats discard variant information, such as allele count (AC), allele frequency, filters, etc. (see Supplementary Section S4) and may therefore be unsuitable for certain tasks. Of the existing file formats, SAVVY, PLINK2 and XSI offer features closest to BCF. Because XSI keeps the variants in BCF format, it is compatible with BCFTools at the variant level. It can also be queried synchronously with VCF/BCF files through the HTSLIB. For example, to query common variant loci between a VCF sample and a XSI reference panel.

### 3.2 Compression

**Fig. 3.**
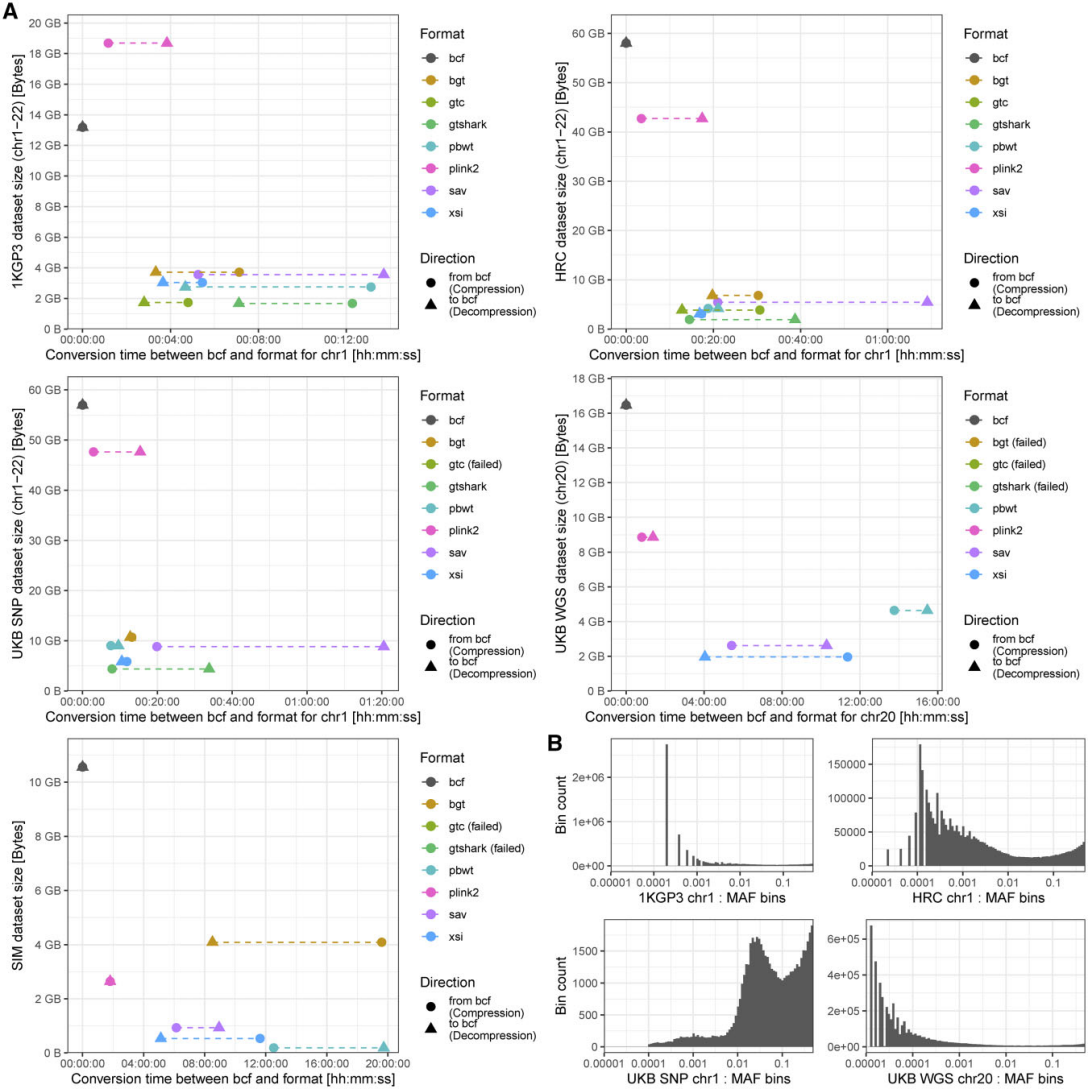
(**A**) File sizes with conversion time to and from BCF on data from the 1000 Genome Project Phase 3, the Haplotype Reference Consortium, the UK Biobank SNP Array genotypes, the UK Biobank WGS genotypes and a 10-Mb simulated region with 1 million samples. (**B**) MAF distribution for the real (non-simulated) datasets

The different file formats have been evaluated for file size and conversion times from and to BCF on all datasets (dataset details in Supplementary Section S1 and commands in Supplementary Section S2). Figure 3 summarizes the results. The methods can be split into three classes: BCF relies on a binary representation of the data followed by standard compression, PLINK2 employs a binary and sparse representation and finally all remaining methods employ data rearrangement (PBWT, sorting) before re-encoding and compressing. Rearrangement-based methods achieve the smallest footprint at the cost of conversion or access time. Overall, XSI achieves between 4× and 20× file size reduction.

The reported decompression times reflect the conversion of a given format to uncompressed BCF (bypassing the gzip compression) in order to demonstrate the format decompression speed and because this is what would be used to pipe into other tools. Piping compressed BCF is an issue because the first tool has to reconvert to BCF and compress it (gzip) and then the second tool will have to decompress the data right after to use it. This chained compression–decompression is an unnecessary and computationally expensive step in an analysis pipeline. SAVVY did not provide the option to output uncompressed BCF and therefore exhibited longer decompression time because it output compressed BCF.

GTC failed to compress the larger datasets with the allocated resources (24 h, 64 GB of memory), BGT would crash when compressing the UKB WGS data. The resulting output files of GTShark for UKB WGS and SIM datasets were corrupted (this issue has been mentioned to the authors). PBWT can achieve small file sizes but does not provide efficient random access on the data and the per variant locus dependency makes it costly to access. PLINK2 uses custom code to encode and decode BCF and is faster compared with other methods that rely on the HTSLIB but exhibits larger file sizes. XSI manages to compress and decompress all datasets, with a competitive file size and access time. Allele frequency bin counts are given for the real datasets in Figure  3B to show the sparsity of the data, low MAF values mean sparse data.

### 3.3 Data access

**Fig. 4.**
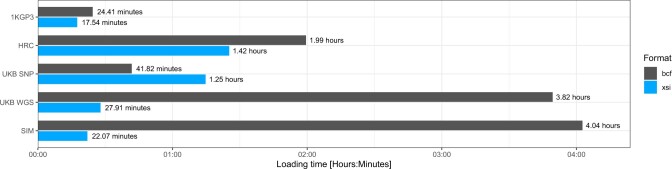
Loading times compared with BCF on real and simulated datasets. Total loading time for chr1-22 on 1KGP3, HRC and UKB SNP. Loading time for chr20 only on UKB WGS

In order to assess the impact of using XSI compressed data inside a software tool, we looked at the time needed to load data in memory from a compressed XSI file against BCF (Fig. 4) and found that the performance depends on the balance between rare and common variants; from 2× slower on UKB SNP array data to 8× faster on UKB WGS and simulated data (Fig. 4). The MAF threshold of XSI can be adapted to improve loading times at the cost of file size (see Supplementary Section S3 and Supplementary Fig. S1). We used the XSI C API we provide to modify SHAPEIT4 (Delaneau *et al.*, 2019) so that genotype data can be directly loaded from XSI files. This significantly speeds-up loading times for WGS data such as the UKB and simulated datasets with minimal coding efforts (changes of less than 10 C/C++ lines of code). For the UK Biobank SNP array, data loading time is slower because it is mostly common variants (see Fig.  3B) and has to perform many costly PBWT rearrangements during access.

**Fig. 5.**
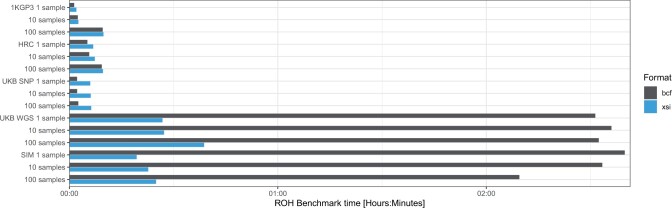
Runtimes of the ROH pipeline XSI decompressed and piped into BCFTools compared with BCF

It is also possible to directly pipe the output of the XSI decompressor into a pipeline that expects BCF. An example is given with a BCFTools run of homozygosity (ROH) pipeline (Narasimhan *et al.*, 2016), once by reading directly from a BCF file and once by redirecting the output of XSI into BCFTools. The analysis was performed on a single chromosome with sample subsets of 1, 10, and 100 samples to assess sample extraction performance as well. Outputs for both methods are identical. The commands for both pipelines are given in Supplementary Section S5. Results are shown in Figure 5 and exhibit similar results to the loading time benchmark except a slightly worse performance for the 1KGP3 and HRC datasets, because the decompressor not only decompresses the data but also converts it back to BCF before writing it into the pipeline. The UKB SNP array dataset suffers from the PBWT rearrangements needed when extracting samples. However, running with the UKB WGS and simulated data, the results are sped-up by a factor of over 5× compared with BCF.

### 3.4 Computation on encoded data

The idea of computing directly on encoded data instead of decoding the data for analysis has been proposed in Loh *et al.* (2012) under the term ‘compressive genomics’ and introduces the concept of ‘algorithms that compute directly on compressed genomic data to allow analyses to keep pace with data generation’. This concept was developed and applied to further bioinformatics fields as ‘compressive acceleration’ (Berger *et al.*, 2016). In order to allow for ‘compressive acceleration’, XSI provides access to the internal data structures. This makes it possible to apply algorithms on encoded data and do computations without full decompression. In order to demonstrate the advantages of ‘compressive’ methods, two example applications are provided. First, computation of ACs for every variant. Second, dot products between encoded genotype data and phenotype data.

#### 3.4.1 Computation of AC (allele frequency)

The computation of AC is done for example each time a BCF is subsetted. We compared updating the fields ‘AC’ (alternative AC) and ‘AN’ (total allele number) with BCFTools (fill-tags plugin) and using the XSI internal representation. Because these statistics do not rely on the ordering of the samples, they can be extracted without the costly PBWT transform and only require us to decompress the zstd layer. The internal sparse and WAH representations allow us to get the counts in a very efficient manner. XSI allowed for a speed-up from 25× to over 1000× (Table 2). Commands are given in Supplementary Section S6.

**Table 2.** Runtimes of the alternative AC and total AC (AN) recomputation with BCFTools (plugin fill-tags) versus XSI

AC/AN Count	bcf fill-tags	xsi	Speed-up
1KGP3 chr1	29m27s	1m09s	>25×
HRC chr1	130m26s	2m59s	>43×
UKB SNP chr1	89m01s	2m19s	>38×
UKB WGS chr20	2464m58s	8m36s	>280×
SIM	2768m37s	2m12s	>1250×

*Note*: Both programs annotate the BCF file with the same results.

Computation and access to these summary statistics is key for many applications. For example, when getting the alternative ACs for all samples of a given population, working with the encoded representations allows us to get the counts orders of magnitude faster than with the BCF plugin.

#### 3.4.2 Dot products

Dot products are at the base of many statistical tests performed in the context of GWAS, the most known being linear regression. We therefore wanted to evaluate the performance of the XSI internal structures for this operation. A benchmark was created to run dot products between genotypes and phenotypes (floating point double values). We compare the traditional array of genotypes (as in BCF) to our PBWT WAH encoded and sparse data structures. A program was written to compute the dot products from genotypes from either a BCF or a XSI file against given phenotype values, which allowed us to compare runtimes as well as the validity of the results.

**Fig. 6.**
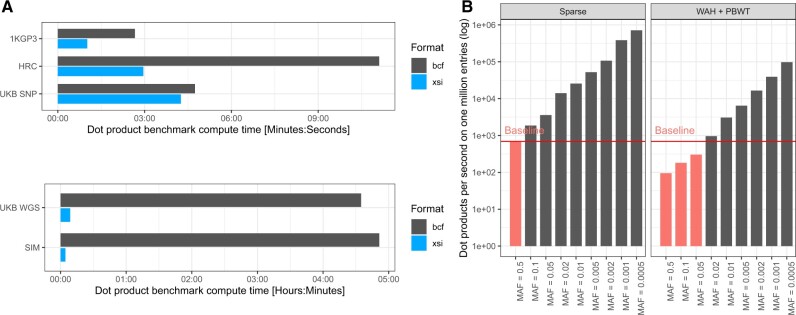
(**A**) Runtimes of the dot product computation between the genotype array and a phenotype array at each variant locus, for all the datasets (single chromosome). (**B**) The number of dot products per second relative to encoding and MAF compared with baseline (plain array). Cases slower than baseline are shown in red.

Figure  6A shows the runtimes on all datasets. This runtime encompasses file loading and decompression (gzip for BCF and zstd for XSI). XSI shows faster runtimes on all datasets. Figure  6B shows a synthetic benchmark (1 million samples) to assess the performance of the structures as a function of MAF compared with a plain array (baseline). The sparse representation only computes values for non-zero (hom ref) data and therefore scales with sparsity (low MAF values). Sparse computation is key to many methods, for example (Mbatchou *et al.*, 2021). PBWT-reordered WAH suffers from non-linear data access patterns and performs worse than baseline for high MAF (>5%). However, lower MAF values still allow to skip portions of zero values and achieve good performances. The MAF distribution inside the dataset (Fig.  3B) will dictate performance. Nevertheless, for all datasets, using XSI and performing computations on the encoded data resulted in a speed-up (30× for UKB WGS) compared with computing the dot product on a plain array (baseline) from BCF, even for the UKB SNP data. The dot product benchmark can outperform the loading time benchmark because data are not decompressed into plain arrays but used directly.

Many software suites make use of the sparse representation when effective. However, they require conversion from the input format to the internal representation, while here it is possible to directly load the file without any conversions and directly work with the encoded data. This does not only speed up computations but file loading as well, which sometimes is the major bottleneck. The WAH representation, even with the cost of PBWT rearrangement, allows our method to achieve further gains for variants with MAF <5%. The increased computation cost for variants with MAF ≥5% is not a major concern because the number of these extremely common variants is limited and will not increase much in the future, newly discovered variants being (very) rare as can be seen in the UKB WGS MAF distribution (Fig.  3B).

## 4 Discussion

In this article, we have presented XSI, a new file format that comes with a command line tool XSI, C API, open-source code and format specifications. XSI was shown to reduce file size footprint, 4–20× times compared with BCF, to improve loading times, up to 8× times faster than BCF and to speed-up computations for analyses on real and simulated datasets. XSI is particularly effective on WGS data such as from the UK biobank and we show the usefulness of computation on the encoded data with 280× faster computation of ACs and 30× faster dot product computation on the UK biobank WGS data. Simulated data show that with future WGS datasets, the difference between BCF and XSI will increase further.

Data generated from population scale projects with WGS created the need for novel file formats in order to scale and reduce costs, not only for data storage and transfer but also for analyses. XSI achieves the goals of providing a smaller footprint, speeding up data access and computations and allows future extensions.

XSI was mainly designed to reduce the footprint of large human genotype collections so it focused on genotype data alone and does not yet support other fields such as genotype likelihoods or dosages. However, because the format is extensible, data blocks for these fields can be added without breaking compatibility with the current version.

Future works include developing specialized compression methods for other fields. Other future works include adapting existing methods to take advantage of XSI internal data structures to improve performance through ‘compressive genomics’, for example, for GWAS or haplotype imputation. Finally, with the smaller file footprint and efficient internal data structures, XSI would also fit computations on accelerators with limited memory such as graphic cards (GPUs) or field programmable gate arrays (FPGAs).

We provide a solution to compress VCF/BCF files, a command line tool, an API for synchronous mixed format reading compatible with HTSLIB and access to inner data structures for fast computation. A structured format approach also allows to expand XSI in the future. Our results show the format to be well suited for storage, access and analysis of existing and future WGS genotype datasets such as from the UK biobank.

## Supplementary Material

btac413_Supplementary_Data
