# Supplementary material for: XSI—a genotype compression tool for compressive genomics in large biobanks
Source: Bioinformatics. 2022 Jun 24;38(15):3778–84. doi: 10.1093/bioinformatics/btac413 (PMC9344850; doi:10.1093/bioinformatics/btac413)
Supplement: btac413_Supplementary_Data [file btac413_supplementary_data.pdf]

XSI - A genotype compression tool for compressive genomics in large biobanks - Supplementary Materials

S1 Datasets

1KGP3 :
Link : https://www.internationalgenome.org/data-portal/data-collection/phase-3

Size chr1-22 (bcf) : 13 GB
Number of vcf lines chr1-22 : 81,271,745
Number of samples : 2,504

HRC :
Link : https://ega-archive.org/studies/EGAS00001001710

Size chr1-22 (bcf) : 55 GB
Number of vcf lines chr1-22 : 39,131,578
Number of haplotypes : 27,165

UKB SNP :
Link : https://www.ukbiobank.ac.uk

Size chr1-22 (bcf) : 54 GB
Number of vcf lines chr1-22 : 670,734
Number of samples : 488,205

UKB WGS :
Link : https://www.ukbiobank.ac.uk

Size chr22 (bcf) : 16.5 GB
Created from the original VCF.gz by filtering out non GT sample fields and converting to compressed BCF with BCFTools
Number of vcf lines chr22 : 12,897,913
Number of samples : 150,119

SIM :
Created with msprime : https://tskit.dev/msprime/docs/stable/intro.html
Size (bcf) : 9.9 GB
Number of vcf lines : 2,271,035
Number of samples : 1,000,000

S2 Compression Commands

bgt https://github.com/lh3/bgt

```
bgt import <output_file> <input_file>
```

gtc https://github.com/refresh-bio/GTC

```
gtc compress -o <output_file> -b <input_file>
```

gtshark https://github.com/refresh-bio/GTShark

```
gtshark compress-db <input_file> <output_file>
```

pbwt https://github.com/richarddurbin/pbwt

```
pbwt -readVcfGT <input_file> -writeAll <output_file>
```

plink2 https://www.cog-genomics.org/plink/2.0/

```
plink2 -bcf <input_file> -make-pgen -out <output_file>
```

sav https://github.com/statgen/savvy (sparse threshold based on author recommendations)

```
sav import -6 -phasing full -sparse-threshold 0.001 -pbwt-field
“GT” <input_file> <output_file>
```

xsi https://github.com/rwk-unil/xSqueezeIt (default MAF threshold 0.1 %)

```
xsqueezeit -c -zstd -f <input_file> -o <output_file>
```

S3 File size and loading time as function of MAF

Impact of MAF threshold on file size and loading time is shown in Fig. S1 on HRC and UKB SNP datasets (both chromosome 1). A lower MAF threshold will result in smaller file sizes at the cost of longer loading times. This parameter allows a trade-off between access speed and size.

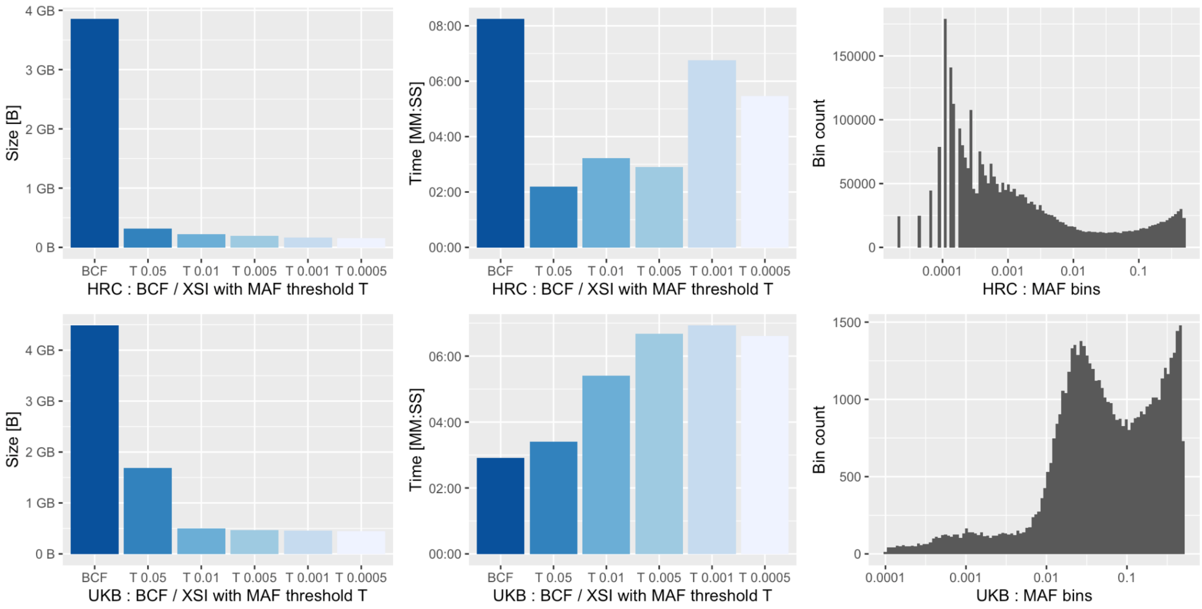

Fig. S1. File size / Loading time relationship to minor allele frequency (MAF) threshold in XSI compression. Minor allele frequency distribution of the variants in the dataset is shown on the right. The minor allele frequency reflects the sparsity of the data. The higher the minor allele frequency (max 0.5) the denser the data.

S4 Features detailed information

- **Keeps variant info (Lossless variants)** In the VCF specification the variant loci have several fields, the contig (or chromosome), the position, the variant ID, the reference and alternative alleles, a quality score, a filter field, and an information string. For most applications these fields are significant and should therefore be conserved. For example variants spanning a larger region require an “end” position in the variant string info. Requests for extraction of variants overlapping a region will not work properly without it.

Input (expected output) :

```
#CHROM  POS     ID      REF     ALT     QUAL    FILTER  INFO    FORMAT  HG00110 HG00111 HG00112 HG00113 HG00114
HG00115 HG00116 HG00117 HG00118 HG00119
20      60810   rs527408846     G       GA      100     PASS
AC=0;AF=0.000798722;AN=20;NS=2504;DP=21358;EAS_AF=0;AMR_AF=0.0058;AFR_AF=0;EUR_AF=0;SAS_AF=0;AA=|||unknown(NO_C
OVERAGE);VT=INDEL     GT      0|0     1|0     0|0     0|0     0|0     0|0     0|0     0|0     0|0     0|0
```

bgt (discards variant information) :

```
#CHROM  POS     ID      REF     ALT     QUAL    FILTER  INFO    FORMAT  HG00110 HG00111 HG00112 HG00113 HG00114
HG00115 HG00116 HG00117 HG00118 HG00119
20      60810   .       G       GA      0       .       .       GT      0/0     1/0     0/0     0/0     0/0
0/0     0/0     0/0     0/0     0/0
```

gtc (discards variant information) :

```
#CHROM  POS     ID      REF     ALT     QUAL    FILTER  INFO    FORMAT  HG00110 HG00111 HG00112 HG00113 HG00114
HG00115 HG00116 HG00117 HG00118 HG00119
20      60810   .       G       GA      0       .       .       GT      0|0     1|0     0|0     0|0     0|0
0|0     0|0     0|0     0|0     0|0
```

pbwt (discards variant information) :

```
#CHROM  POS     ID      REF     ALT     QUAL    FILTER  INFO    FORMAT  HG00110 HG00111 HG00112 HG00113 HG00114
HG00115 HG00116 HG00117 HG00118 HG00119
20      60810   .       G       GA      .       PASS    AC=1;AN=20      GT      0|0     1|0     0|0     0|0
0|0     0|0     0|0     0|0     0|0     0|0
```

other methods show the same output as input (expected output)

- **Mixed ploidy support** For example on chromosome X for a given variant locus some samples are haploid while others are diploid. The compression method should conserve this information and not transform haploid genotypes into diploid for example.

Input (expected output) mixed ploidy :

```
X       12000041 .      C       A       100     PASS
AC=12;AF=0.00317881;AN=3775;NS=2504;DP=12993;AMR_AF=0.0038;AFR_AF=0.01;EUR_AF=0;SAS_AF=0;EAS_AF=0;AA=C|||;VT=SN
P       GT      0       0|0     0|0     0|0     0       0|0     0       0       0|0     0       0
        0       0|0     0|0     0       0       0       0       0       0       0|0     0       0|0
        0|0     0|0     0|0     0|0     0       0|0     0|0     0       0|0     0       0|0     0|0
        0       0|0     0       0       0       0
```

bgt :

```
X       12000041 .      C       A       0       .       .       GT      0/0     0/0     0/0     0/0
        0/0     0/0     0/0     0/0     0/0     0/0     0/0     0/0     0/0     0/0     0/0     0/0
        0/0     0/0     0/0     0/0     0/0     0/0     0/0     0/0     0/0     0/0     0/0     0/0
        0/0     0/0     0/0     0/0     0/0     0/0     0/0     0/0     0/0     0/0     0/0     0/0
        0/0
```

gtc :

```
X       12000041 .      C       A       0       .       .       GT      0/.     0|0     0|0     0|0
        0/.     0|0     0/.     0/.     0|0     0/.     0/.     0/.     0|0     0|0     0/.     0/.
        0/.     0/.     0/.     0/.     0|0     0/.     0|0     0|0     0|0     0|0     0|0     0/.
        0|0     0|0     0/.     0|0     0/.     0|0     0|0     0/.     0|0     0/.     0/.     0/.
        0/.
```

gtshark :

```
X       12000041 .      C       A       100     PASS
AC=12;AF=0.00317881;AN=3775;NS=2504;DP=12993;AMR_AF=0.0038;AFR_AF=0.01;EUR_AF=0;SAS_AF=0;EAS_AF=0;AA=C|||;VT=SN
P       GT      0|2     0|0     0|0     0|0     0|2     0|0     0|2     0|2     0|0     0|2     0|2
        0|2     0|0     0|0     0|2     0|2     0|2     0|2     0|2     0|2     0|0     0|2     0|0
        0|0     0|0     0|0     0|0     0|2     0|0     0|0     0|2     0|0     0|2     0|0     0|0
        0|2     0|0     0|2     0|2     0|2     0|2
```

pbwt (would not compress)

plink :

```
X       12000041 .      C       A       100     PASS
AC=12;AF=0.00317881;AN=3775;NS=2504;DP=12993;AMR_AF=0.0038;AFR_AF=0.01;EUR_AF=0;SAS_AF=0;EAS_AF=0;AA=C|||;VT=SN
P       GT      0|0     0|0     0|0     0|0     0|0     0|0     0|0     0|0     0|0     0|0     0|0
        0|0     0|0     0|0     0|0     0|0     0|0     0|0     0|0     0|0     0|0     0|0     0|0
        0|0     0|0     0|0     0|0     0|0     0|0     0|0     0|0     0|0     0|0     0|0     0|0
        0|0     0|0     0|0     0|0     0|0     0|0
```

Other methods show the same output as input (expected output).

- **Keeps phase information** This is the specific phasing of two or more genotypes for a given locus. For haploid samples this doesn't matter but for diploid (or polyploid) samples the VCF specifications allow to represent unphased "0/1" and phased "0|1" genotypes. Often all samples at every variant are either phased or unphased but it is possible to have unphased samples with phased samples or partially phased samples in the same VCF file. This occurs when merging different datasets for example. The phasing information should not be lost or modified.

Input (expected output) one unphased sample all other phased :

```
#CHROM  POS     ID      REF     ALT     QUAL    FILTER  INFO    FORMAT  HG00110 HG00111 HG00112
HG00113 HG00114 HG00115 HG00116 HG00117 HG00118 HG00119
20      60343   rs527639301     G       A       100     PASS
AC=0;AF=0.000199681;AN=20;NS=2504;DP=20377;EAS_AF=0;AMR_AF=0.0014;AFR_AF=0;EUR_AF=0;SAS_AF=0;AA=.|||;VT=SNP
        GT      0/0     1|0     1|1     0|0     0|0     0|0     1|0     0|1     0|0     0|0
```

bgt (outputs everything as unphased) :

```
#CHROM  POS     ID      REF     ALT     QUAL    FILTER  INFO    FORMAT  HG00110 HG00111 HG00112
HG00113 HG00114 HG00115 HG00116 HG00117 HG00118 HG00119
20      60343   .       G       A       0       .       .       GT      0/0     1/0     1/1     0/0
        0/0     0/0     1/0     0/1     0/0     0/0
```

gtc (outputs everything as phased) :

```
#CHROM  POS     ID      REF     ALT     QUAL    FILTER  INFO    FORMAT  HG00110HG00111    HG00112
HG00113 HG00114 HG00115 HG00116 HG00117 HG00118 HG00119
20      60343   .       G       A       0       .       .       GT      0|0     1|0     1|1     0|0
        0|0     0|0     1|0     0|1     0|0     0|0
```

gtshark (outputs everything as phased) :

```
#CHROM  POS     ID      REF     ALT     QUAL    FILTER  INFO    FORMAT  HG00110HG00111    HG00112
HG00113 HG00114 HG00115 HG00116 HG00117 HG00118 HG00119
20      60343   rs527639301     G       A       100     PASS
AC=0;AF=0.000199681;AN=20;NS=2504;DP=20377;EAS_AF=0;AMR_AF=0.0014;AFR_AF=0;EUR_AF=0;SAS_AF=0;AA=.|||;VT=SNP
        GT      0|0     1|0     1|1     0|0     0|0     0|0     1|0     0|1     0|0     0|0
```

pbwt (outputs everything as phased) :

```
#CHROM   POS      ID       REF      ALT      QUAL     FILTER   INFO     FORMAT   HG00110HG00111   HG00112
HG00113  HG00114  HG00115  HG00116  HG00117  HG00118  HG00119
20       60343    .        G        A        .        PASS     AC=5;AN=20        GT      0|0      1|0      1|1
         0|0      0|0      0|0      1|0      0|1      0|0      0|0
```

plink2 (outputs everything as phased) :

```
#CHROM   POS      ID       REF      ALT      QUAL     FILTER   INFO     FORMAT   HG00110HG00111   HG00112
HG00113  HG00114  HG00115  HG00116  HG00117  HG00118  HG00119
20       60343    rs527639301       G        A        100      PASS
AC=0;AF=0.000199681;AN=20;NS=2504;DP=20377;EAS_AF=0;AMR_AF=0.0014;AFR_AF=0;EUR_AF=0;SAS_AF=0;AA=.|||;VT=SNP
         GT       0|0      1|0      1|1      0|0      0|0      0|0      1|0      0|1      0|0      0|0
```

Other methods show the same output as input (expected output).

- **Multi-Allelic site support** For a given reference allele at a given locus there can be multiple variants. For example one mutation in the population could be A->T and another A->C. The VCF format allows for multiple alternate alleles per locus. If there are more than two (reference, alt) possible alleles (reference, alt1, alt2, …,altn). Formats that don't support multi-allelic sites will split them, so instead of having a single VCF line for the locus with multiple alt alleles, there will be one VCF line per alt allele.

- **Sample extraction** The sample extraction feature provides the same functionality as bcftools view --samples "NA12878,HG001" but on the compressed file format directly and allows to output a VCF/BCF with only the requested samples.

- **Region extraction** The region extraction feature provides the same functionality as bcftools view --regions "chromosome:start_position-end_position" but on the compressed file format and allows to output a VCF/BCF with only the variants that overlap the requested region.

- **Simultaneous access with VCF files through HTSLIB "sync_bcf_reader"** This reader is part of the HTSLIB [ref] and makes it possible to go through multiple different files in a synchronous manner. It allows to keep multiple VCFs open and stream them using a "next_line" iterator in a seamless manner without worrying about chromosomes and site synchronization. This is very useful to parse an input VCF file against a reference panel VCF collection. By keeping the variant information in the BCF/VCF format it allows querying multiple files through this iterator with HTSLIB. Being compatible allows for seamless integration of mixed file format queries with HTSLIB. Any file format that keeps the variant information in VCF/BCF (e.g., XSI, BGT, and GTC) could be queried through this iterator. However, the file format should also provide a C interface similar or equivalent to HTSLIB to retrieve the genotype data when the reader retrieves a line.

S5 Run of Homozygosity pipeline

BCFTools Run of Homozygosity command :

```
bcftools roh -G30 --AF-dflt 0.4 chr1.bcf -S "sample_list_file"
```

The command modified to work with XSI is :

```
xsqueezeit  -x -f chr1.xsi -S "sample_list_file" | bcftools roh
-G30 --AF-dflt 0.4
```

S6 AC/AN Computation

BCFTools plugin “fill-tags” :

```
bcftools +fill-tags <input_file>.bcf -o <output_file>.bcf -Ob --
-t AN,AC"
```

XSI :

```
af_stats -f <input_file>.xsi -o <output_file>.bcf
```
